# Supplementary material for: An Easy-to-Use Prehospital Indicator to Determine the Severity of Suspected Heat-Related Illness: An Observational Study in the Tokyo Metropolitan Area
Source: Diagnostics (Basel). 2023 Aug 15;13(16):2683. doi: 10.3390/diagnostics13162683 (PMC10452966; doi:10.3390/diagnostics13162683)
Supplement: Supplementary file 1 [file diagnostics-13-02683-s001.zip › diagnostics-2475617-supplementary.pdf]

diagnostics

MDPI

Supplementary Material: An Easy-to-use Prehospital Indicator to Determine the Severity of Suspected Heat-related Illness: An Observational Study in the Tokyo Metropolitan Area

Junko Yamaguchi, Kosaku Kinoshita and Minami Takeyama

Table S1. Classification of the level of consciousness of our study population.

a. JCS and Mayo classification conversion.

JCS	Mayo Clinic Classification	Alert or Not-Alert
300,200	Deep coma	Not-Alert
100	Semi coma	Not-Alert
10,20	Somnolence	Not-Alert
1,2,3	Confusion	Not-Alert
None	Alert	Alert

b. Mayo Clinic Classification-based stratification of the study population.

	All (n=2528)	Non-S group (n=2468)	S group (n=60) [1]	p-value[2]
Alert	1482/2528 (58.6%)	1481/2468 (60.0%)	1/60 (1.7%)	<0.001
Confusion	946/2528 (37.4%)	923/2468 (37.4%)	60/23 (38.3%)	0.882
Somnolence	60/2527 (2.37%)	49/2468 (1.99%)	11/60 (18.3%)	<0.001
Stupor	5/2528 (0.2%)	3/2468 (0.12%)	2/60 (3.33%)	<0.001
Semi coma	8/2527 (0.32%)	3/8 (37.5%)	5/8 (62.5%)	<0.001
Deep coma	27/2528 (1.1%)	9/2468 (0.36%)	18/60 (30.0%)	<0.001

The Japan Coma Scale score (JCS) has found wide application in the assessment of the patient's level of consciousness in the prehospital setting in Japan. Based on the JCS scores, the level of consciousness has been classified into four main categories: Alert (0) and 1-digit, 2-digit, and 3-digit codes based on an eye response test, wherein each of these categories has three subcategories. However, the Glasgow Coma Scale (GCS) has not been used in the prehospital care setting in Tokyo, Japan. Therefore, it was difficult to convert the JCS evaluation into a GCS score because of the different evaluation methods that are used in these two scales. Therefore, in this study, we converted the level of consciousness ascertained using the JCS to a Mayo Clinic Classification (Mayo Clinic and Mayo Fundation: Clinical Examination in Neurology, pp. 201–04, W.B. Sanders Company, Philadelphia, 1964), which has been presented in Table S1b: Mayo Clinic Classification-based stratification of the study population. We then included the result as an explanatory variable (Alert or Not-Alert) in the multiple logistic regression analysis. Therefore, the study population was divided into two groups based on the classification of illness severity as either life-threatening or non-life-threatening. We excluded patients with out-of-hospital cardiopulmonary arrest and, therefore, Grades 1 and 2, non-S group; Grades 3 and 4, S group[1]; and Grade 5 were not considered. The chi-square test was performed[2].

Table S2. Comparison of patient characteristics (prehospital setting) between the two groups using univariate analysis[a].

Characteristic	All (n=2528)	Non-H group (n=1484)	H group (n=1044)	*p*-value[b]
Age (years) mean±SD (min, max)	62.8±21.7 (20, 101)	58.3±21.9 (20, 99)	69.2±19.7 (20, 101)	<0.0001

Sex (M:F) [M/total (%)]	1532:996 [60.6]	899:585 [60.6]	633:411 [60.6]	0.98
Systolic BP (mmHg)	126.6±31.6	126.2±25.0	127.2±26.6	0.24
Diastolic BP (mmHg)	71.2±15.2	72.0±15.1	70.1±15.4	0.003
Heart rate (bpm)	95±20.8	91.6±19.4	98.7±22.0	<0.0001
Respiratory rate (bpm)	20±3.92	19.8±3.64	20.3±4.26	<0.001
Body temperature (°C)	37.1±1.2	36.8±0.89	37.6±1.34	<0.0001
Location (indoor:outdoor) [indoor/total (%)]	1483:1045 [58.7]	818:666 [55.1]	665:379 [63.7]	<0.0001
Older adults (older adults:non-older adults) [older /total (%)]	1433:1095 [56.7]	577:907 [38.9]	646:398 [61.9]	<0.0001
Consciousness level (alert:not alert) [alert/total (%)]	1481:1047 [58.6]	1050:434 [70.1]	431:613 [41.3]	<0.0001

Data are shown as the mean ± standard deviation, or n (%)
[a] The study population was divided into two groups based on the classification of illness severity to identify the severity of requirement for hospitalization (H group) or non-requirement for hospitalization (non-H group).
Grade 1, does not require hospitalization; Grade 2, necessitates hospitalization, but is not life-threatening; Grade 3, considered to be life-threatening; Grade 4, critical illness with impending danger of death; and Grade 5, death. Grade 1, non-H group; Grades 2–4, H group; Grade 5 was not considered, as we excluded patients with out-of-hospital cardiopulmonary arrest. Axillary body temperatures were measured. Patients older than 70 years were classified as older patients in this study. The classification of the level of consciousness was divided into two categories: alert or not alert.
[b] The Mann–Whitney U test and chi-square test were conducted.
Abbreviations: BP, blood pressure; H, hospitalized; M/F, male/female; non-H, non-hospitalized.

Table S3. Results of multivariate logistic regression analysis of factors associated with the severity of heat-related illness (prehospitalization setting).

Factor	Correlation coefficient	SE	*p*-value	Odds ratio	95% CI
RR >24/min	1.207	0.317	<0.0001	3.34	1.80–6.22
HR >120/min	1.057	0.310	0.001	2.88	1.57–5.29
BT >38.6°C	2.053	0.338	<0.0001	7.79	4.02–15.1
Alert or not	-3.645	1.017	<0.0001	0.026	0.004–0.192
Constant	4.113	1.031	<0.0001	61.125	

Explanatory variables: older adults, respiratory rate, heart rate, temperature, and conscious level.
Older adults: Patients older than 70 years were classified as older patients in this study.

Table S4. Validation of the prediction model （Severity of heat-related illness）.

Distribution	Values	Training cohort	Test cohort
In case of training: test=4:1[*]	AUC (95%CI)	0.9296 (0.892-0.960)	0.9298(0.843-0.981)
	p-values[***]	0.369	
In case of training: test=2:1[**]	AUC (95%CI)	0.9174 (0.867-0.949)	0.9501 (0.902-0.978)
	p-values[***]	0.568	

[*]In total patients enrolled in the first two-thirds of the study period were selected for the training group (n=1618) and the remaining patients in the last third (n=809) were selected for the test group
[**]In total patients enrolled in the first four-fifth of the study period were selected for the training group (n=1941) and the remaining patients in the last fifth (n=486) were selected for the test group
[***] p-values in the Hosmer-Lemeshow good fit test, p>0.05 was used for the cut-off value.
In both distribution method studies, the 95% confidence intervals of the AUC did not differ significantly from the predictive model equation（AUC=0.93,95%CI;0.90-0.96）.

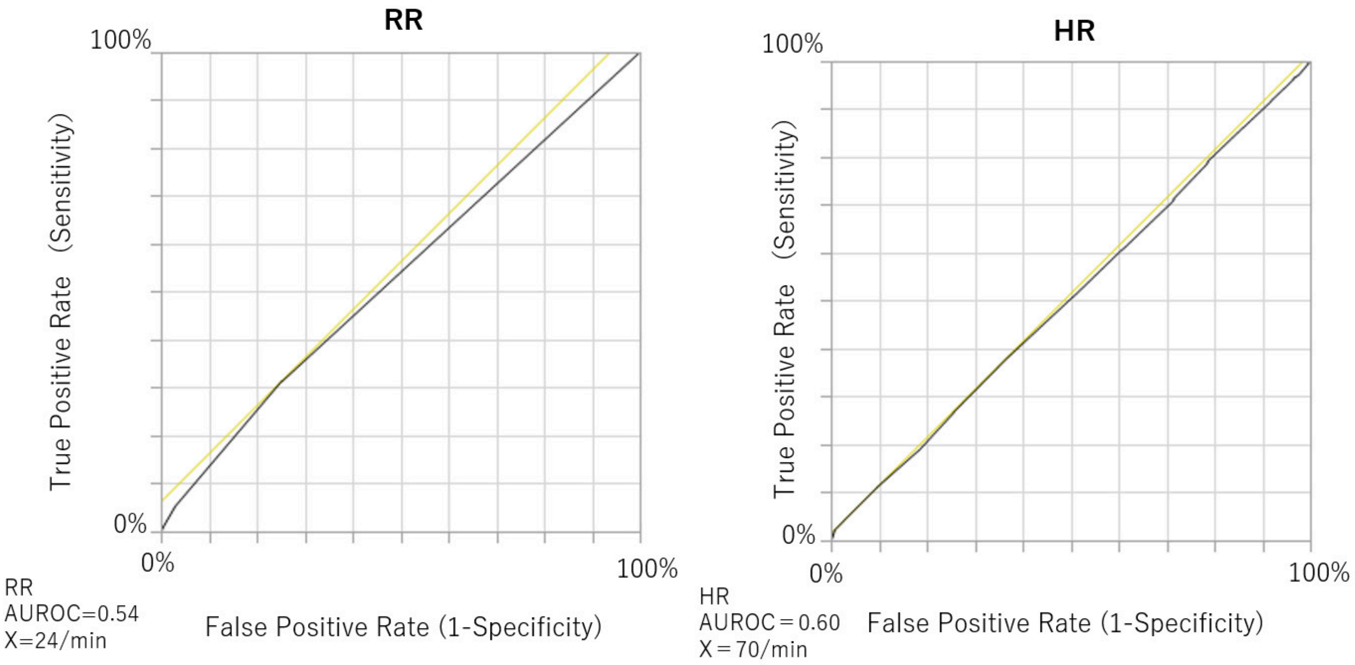

(a)

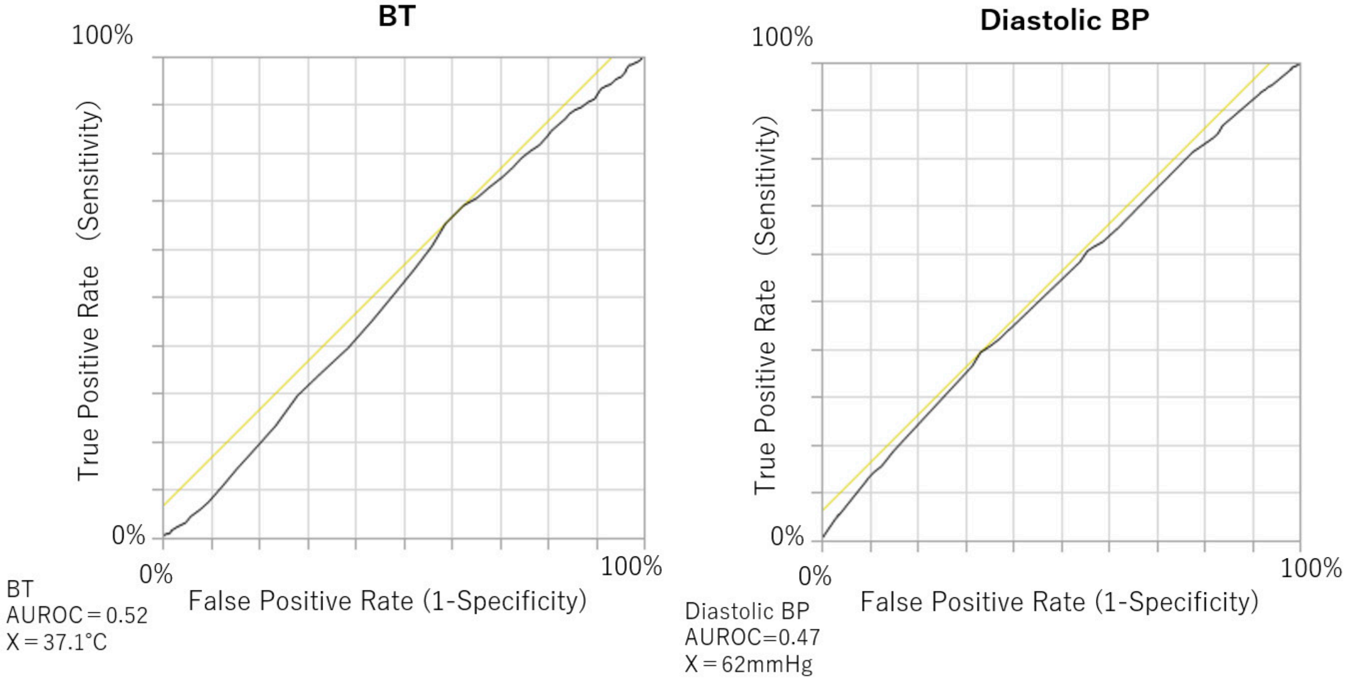

(b)

Figure S1. Receiver operating characteristic (ROC) curves for request of hospitalization in patients with heat-related illness in the prehospitalization setting. The highest AUROC [95% CI] were observed for: **(a)** RR>24 /min (0.54 [0.51–0.56]), HR >70/min (0.60 [0.57–0.62]); (b) BT >37.1°C (0.52 [0.50–0.54]), and diastolic BP >62mmHg (0.47 [0.44–0.49]).

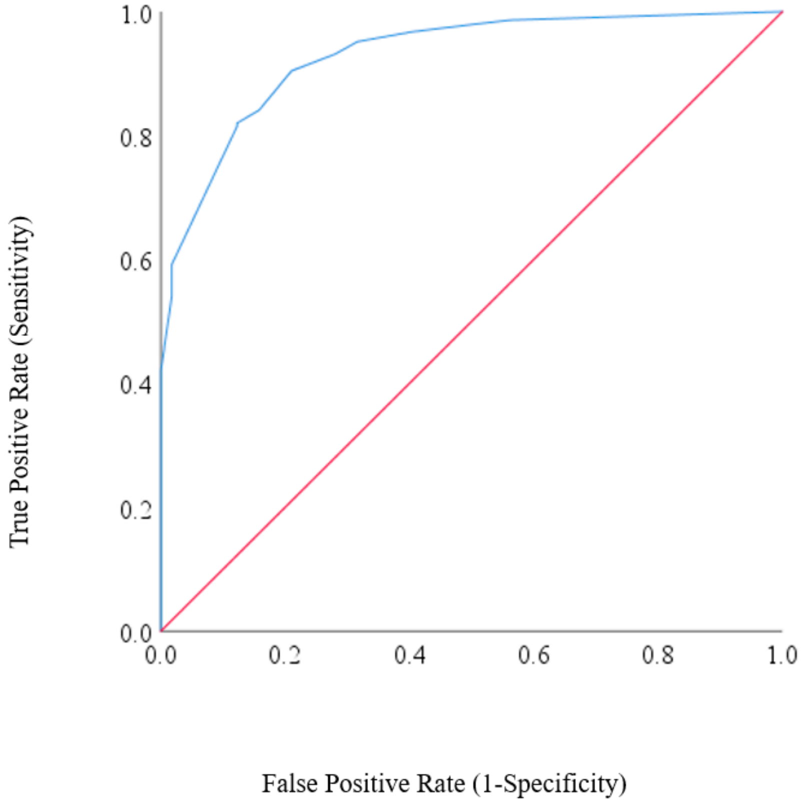

Figure S2. Evaluation of Predictive Models with ROC Curves. The highest areas under the receiver operating characteristic curves (AUROC) were 0.929, with a 95% confidence interval of 0.900-0.958: with a sensitivity of 0.820 and a specificity of 0.877.

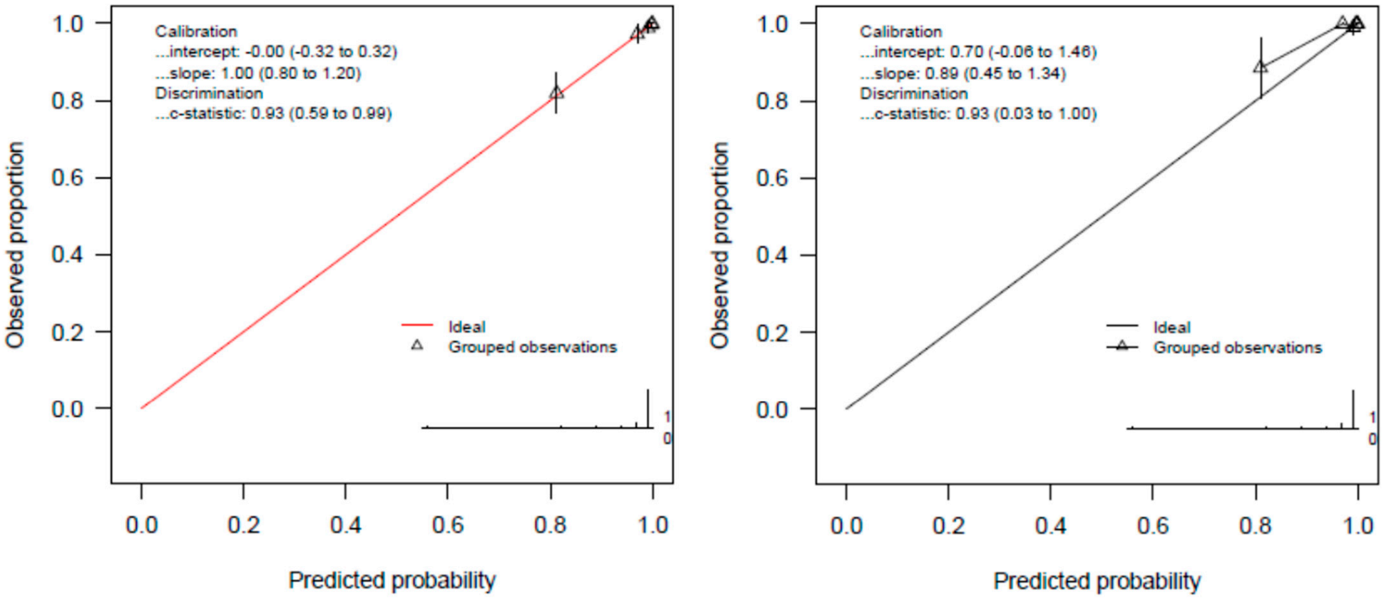

(a) **(b)**

Figure S3. Calibration curve of the nomogram to predict severe heat-related illness in case of splitting the patient data of our study randomly 4:1 (training: test= 4:1). In total patients enrolled in the first four-fifth of the study period were selected for the training group (n=1941) and the remaining patients in the last fifth (n=486) were selected for the test group. **(a)** Calibration curve of the nomogram in the training group; **(b)** Calibration curve of the nomogram in the test group.
Calibration curves depict the calibration of each group in terms of the agreement between the

predicted probability of severe heat-related illness and observed frequency. The "ideal" line indicates the ideal nomogram reference line. The "grouped observations" line was calculated directly from the data set to represent the performance of the nomogram. The C-index for the prediction nomogram via bootstrapping validation was 0.93(95%CI,0.59-0.99) for the training group. As external validation, the C-index for the prediction nomogram was 0.93(95%CI,0.03-1.00) for the test group.

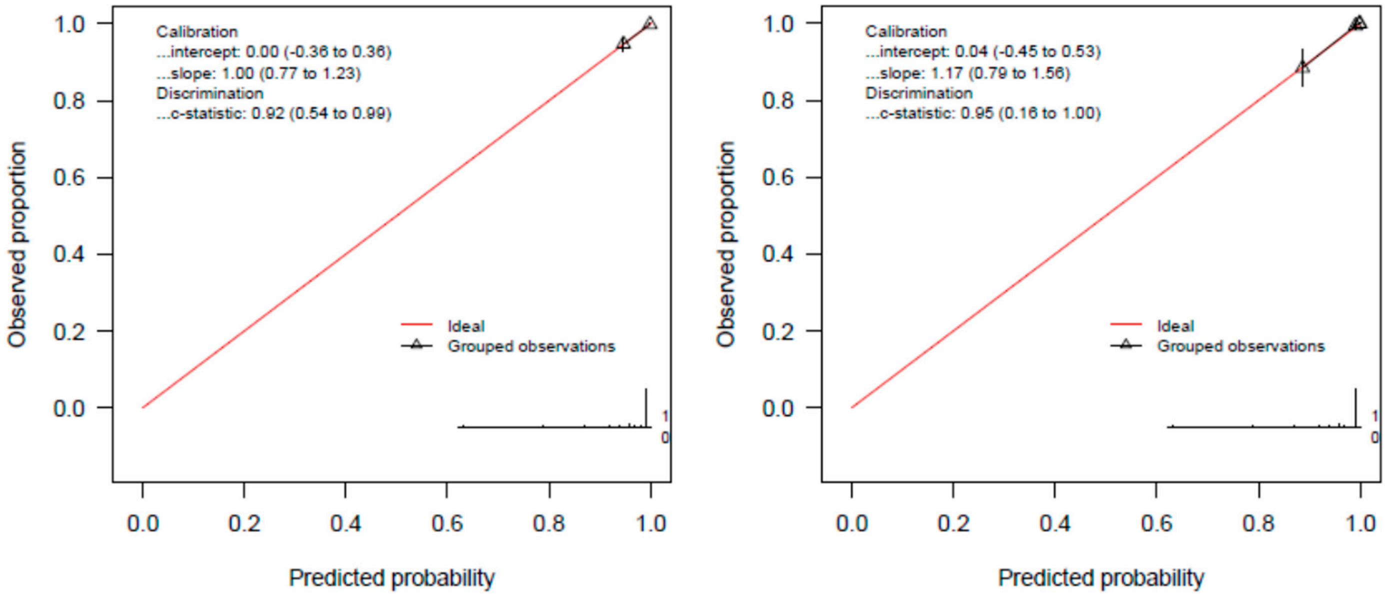

(a) **(b)**

Figure S4. Calibration curve of the nomogram to predict severe heat-related illness in case of splitting the patient data of our study randomly 2:1 (training: test= 2:1). In total patients enrolled in the first two-thirds of the study period were selected for the training group (n=1618) and the remaining patients in the last third (n=809) were selected for the test group. **(a)** Calibration curve of the nomogram in the training group; **(b)** Calibration curve of the nomogram in the test group. Calibration curves depict the calibration of each group in terms of the agreement between the predicted probability of severe heat-related illness and observed frequency. The "ideal" line indicates the ideal nomogram reference line. The "grouped observations" line was calculated directly from the data set to represent the performance of the nomogram. The C-index for the prediction nomogram via bootstrapping validation was 0.92(95%CI,0.54-0.99) for the training group. As external validation, the C-index for the prediction nomogram was 0.95(95%CI,0.16-1.00) for the test group.
